# Prevalence of type 2 diabetes (T2D) in Lebanon: association with inflammatory and infectious clinical markers

**DOI:** 10.1186/s12889-023-17328-6

**Published:** 2023-12-16

**Authors:** Pia Chedid, Elie Salem Sokhn

**Affiliations:** 1 Molecular Testing Laboratory, Medical Laboratory Department, Faculty of Health Sciences, Beirut Arab University, Beirut, Lebanon; 2 Laboratory Department, Lebanese Hospital-Geitaoui University Medical Center, Beirut, Lebanon

**Keywords:** HbA1c, Type 2 diabetes, Prevalence, Inflammation, Infection, Clinical markers

## Abstract

**Background:**

Diabetes is a growing health concern in the Middle East, particularly in countries with high rates of obesity and unhealthy lifestyles. Therefore, this study aimed to determine the prevalence of type 2 diabetes (T2D) in Lebanon and its association with clinical markers of inflammation and infection.

**Methods:**

This cross-sectional study examined retrospectively the medical laboratory record of 4093 patients from all Lebanese regions.

Prevalence of T2D and its association with age, gender, calcium, vitamin D (VitD), neutrophils-to-lymphocytes ratio (NLR), and C-reactive protein (CRP) were determined. The prevalence of infection in a subpopulation of 712 patients tested from blood, body fluid, sputum, swab, tissue, and urine samples and its etiology was also assessed.

**Results:**

Overall, 17% (*n* = 690) of our participants had T2D, and the mean HbA1c was 5.9% ± 1.2. Age, gender, triglycerides, NLR, and calcemia were significantly associated with T2D. The prevalence of infections in a subgroup of 712 patients was 11.1% (*n* = 79). Urinary tract infections (UTIs) caused by *Escherichia coli* (*E. coli*) were the most common cause of infection, with the highest prevalence in the pre-diabetic group. Serum CRP level was significantly higher in the diabetic group than the pre-diabetic and control groups. Diabetic patients also presented a significantly higher percentage of NLR > 3 compared to the pre-diabetic and control groups.

**Conclusion:**

The prevalence of T2D is increasing in the Lebanese population compared to prior reports. These results should be considered to guide effective public health preventive strategies.

## Introduction

Diabetes is the major endocrinopathy of the last century [1], and various factors, including lifestyle changes, have increased the frequency of this disease [2]. Physical activity level, smoking habits, alcohol consumption, and dietary habits are all lifestyle factors connected to diabetes incidence or risk factors associated with diabetes [2]. In addition, diabetes increases the risk of certain diseases, such as cardiovascular disease, kidney and eye diseases, and infectious diseases [1, 3]. The pathophysiology of diabetes involves a complex interplay of various mechanisms. The two main types of diabetes, type 1 and type 2, have distinct underlying causes. While type 1 diabetes is an early-onset autoimmune disease associated with genetic predisposition [4], T2D often develops in individuals over 45 years old and is characterized by insulin resistance and relative insulin deficiency [5]. The pre-diabetic stage of the disease is mainly characterized by the decreased response of target cells to insulin (insulin resistance), excessive glucose production by the liver, and a low-grade chronic inflammation [6]. Risk factors for pre-diabetes include obesity, a sedentary lifestyle, a family history of diabetes, high blood pressure, high triglyceride levels, low HDL cholesterol levels, and a history of gestational diabetes [6]. However, pre-diabetes is still a reversible condition [6]. The progression to T2D is conditioned by the pathologic progression of beta cell dysfunction [5]. At first, increased insulin production compensates for insulin resistance. However, hyperinsulinemia leads overtime to beta cell exhaustion resulting in hyperglycemia; that is when the transition from pre-diabetes to T2D occurs [5]. In 2015, around 415 million adults with diabetes were reported [1], and this number is predicted to increase to 642 million by 2040 [7]. Precisely in the Middle East and North Africa region (MENA), the prevalence of diabetes was 12.2% in 2019 [5], and this percentage is expected to witness a 96% increase by 2045 [8]. Previous reports on limited sample size in Lebanon have shown that the prevalence of T2D is around 15% [9]. According to the American Diabetes Association, the diagnosis of diabetes relies on glycated hemoglobin (HbA1c) as a substitute for fasting blood glucose [7], and HbA1c is a significant indicator of long-term glycemic control with the ability to measure the average blood sugar levels over the past three months [10].

Growing evidence continuously shows that elevated levels of inflammatory markers and some blood tests are associated with an increased risk of diabetes [11, 12]. Several inflammatory markers are commonly elevated in individuals with T2D. These markers can contribute to insulin resistance and other complications associated with the condition. Inflammation plays a crucial role in the development and progression of atherosclerosis, a critical factor in the increased risk of cardiovascular disease in individuals with diabetes [13]. Clinical markers of inflammation, particularly C-reactive protein (CRP), a protein produced by the liver in response to inflammation, can indicate acute or chronic inflammation and are often used to monitor T2D diabetes and cardiovascular diseases by assessing the presence and severity of inflammation [14, 15]. Interestingly, numerous studies have established a strong correlation between inflammation and cardiovascular complications in T2D. In individuals with T2D, the anti-inflammatory effect of insulin-sensitizing treatment has been associated with reduced CRP levels and reduced cardiovascular risk [16]. In addition, high HbA1c has also been viewed as an independent risk factor for stroke and coronary heart disease [10]. In a large meta-analysis examining data from multiple prospective studies, elevated levels of CRP, interleukin-6 (IL-6), and fibrinogen were associated with an increased risk of major cardiovascular events, including heart attacks and strokes in both diabetic and non-diabetic populations [17]. Other studies have shown that elevated levels of CRP, neutrophil lymphocyte ratio (NLR), cholesterol, triglyceride, and calcium are significantly associated with an increased risk of diabetes [12, 18–21]. NLR can provide insights into infection risk and severity in T2D. An elevated NLR suggests an imbalance between neutrophils, typically associated with acute inflammation and infection, and lymphocytes, which play a vital role in the adaptive immune response. A higher NLR can indicate an exaggerated inflammatory response or an impaired adaptive immune response. However, the relationship between NLR and infection risk is complex and can vary based on individual characteristics and the specific type of infection being considered. In 2020, researchers found that the NLR was a predictor of the type of bacterial urinary tract infections (UTIs) in T2D patients, with T2D patients with blood NLR ≥ 3.5 significantly at higher risk for Extended-spectrum ß-lactamase (ESBL)-producing *Escherichia coli* UTIs than ESBL-producing *Klebsiella pneumoniae* UTIs [22].

Furthermore, UTIs are the most common among diabetic patients [14], and several factors, such as weakening of white blood cells, immune system disorders, and bladder dysfunction, can cause UTIs in diabetic patients [1]. According to the literature, systemic inflammatory response is present in UTIs, and in that case, abnormal blood test results, including elevated CRP, high erythrocytes sedimentation rate, and leukocytosis, may be present [23]. It is noteworthy to mention that the prevalence of UTIs among diabetic patients ranges from 8%–26% [1, 24].

Therefore, this study aimed to determine the prevalence of T2D in Lebanon and examine the effect of HbA1c on various clinical markers to evaluate its contribution to systemic inflammation and the risk of urinary tract infection.

## Materials and methods

### Study population

This retrospective cohort study was conducted at three different Lebanese hospitals; the Lebanese Hospital Geitaoui University Medical Center in Beirut, the governmental hospital of Mount Lebanon, Sibline, and a private hospital in the North. Data was collected from medical laboratory records after obtaining the IRB approval from the Lebanese Hospital Geitaoui-University Medical Center (2023-IRB-03). In the case of multiple data from the same participant, repeated measurements were excluded, and only the first entry was included in the analysis. We also excluded participants younger than 18 years old. Therefore, a total of 4,093 adult participants records attending the medical laboratory department between January 2021 and December 2021 were analysed.

### Data collection

The lipid profile, triglycerides, Vitamin D (VitD), CRP, and calcium were conducted with blood serum using Roche Cobas 6000, and the complete blood count results were obtained using Pentra DF Nexus automated hematology analyzer. In addition, a high-performance liquid chromatographic (HPLC) method (G7 HPLC Analyzer) was used to determine the HbA1c.

HbA1c levels were used to classify the participants according to three different groups; non-diabetic; participants with HbA1c levels < 5.7%, pre-diabetic participants with HbA1c levels ≥ 5.7% and ≤ 6.5%, and diabetic group; patients with HbA1c levels > 6.5%.

A subpopulation of 712 patients were collected from various sites, including blood, body fluids, sputum, swabs, tissues, and urine. Gram-negative isolates included in this study were identified using API 20E and API 20NE system. Additionally, gram-positive isolates were identified using catalase and coagulase tests and the BD Phoenix automated system.

In addition, the age, gender, body mass index (BMI), and region of each patient were analysed.

### Statistical analysis

Statistical analyses were performed using SPSS statistical software version 24.0 (SPSS, Inc, Chicago, Illinois). Continuous variables were presented as mean values ± standard deviations, and categorical variables were given as frequencies and percentages. Normality was tested using the Shapiro–Wilk test. The Chi-square test (χ2) was performed for categorical variables analysis. Alternatively, the analysis of variance (ANOVA) was applied to continuous variables that followed the normal distribution. The significance level was set at P ≤ 0.05.

To test the association with diabetes, a binary univariate logistic regression model was first used. The variables that showed significant associations (*P* < 0.01) were further included in the multivariate analysis. The independent variables were: age, gender, BMI, triglycerides, NLR, and calcemia. Effect sizes with 95% confidence intervals were calculated. This analysis was performed under the assumption of an additive model.

Sample size calculation (https://clincalc.com/stats/samplesize.aspx) showed a statistical power of at least 0.8 in a two-sided test with *P* = 0.05.

## Results

Our study encompasses 4,093 Lebanese individuals with different demographic, biological, and infectious variables, all shown in Table 1. The mean age of participants was 58.2 (± 16.2) years old (Table 1). Female participants accounted for 55.8% of the population, and male participants for 44.2% (Table 1). Participants mostly came from Mount Lebanon and Beirut regions (45.1% and 41.2%, respectively in Table 1), followed by North of Lebanon (6.7%), Bekaa (4.3%), and South of Lebanon (2.7%). In contrast with BMI and HbA1c levels (34.5 ± 7.9 kg⁄m2 and 5.9% (± 1.2) respectively in Table 1), the lipid profile, the differentiated complete blood count, VitD, and CRP levels were all in the reference range (Table 1). Among patients tested for infections from blood, body fluid, sputum, swab, tissue, and urine samples (*n* = 712), 11.1% (*n* = 79) tested positive (Table 1).

**Table 1:** Characteristics, samples and infection status of the study participants

Characteristics	Participants (*n* = 4,093)
**Age (years)**	58.2 ± 16.2
**Gender**	
*Female (%)*	2284 (55.8%)
*Male (%)*	1810 (44.2%)
**Region**	
*Beirut*	1687 (41.2%)
*Mount Lebanon*	1845 (45.1%)
*South of Lebanon*	110 (2.7%)
*North of Lebanon*	274 (6.7%)
*Bekaa*	178 (4.3%)
**BMI (kg/m**^**2**^**)**	34.5 ± 7.9
**HBA1C (%)**	5.9 ± 1.2
**Total Cholesterol (mg/dL)**	184.4 ± 42.8
**HDL-C (mg/dL)**	51.9 ± 15.3
**LDL-C (mg/dL)**	105.3 ± 36.0
**Triglycerides (mg/dL)**	144.9 ± 101.4
**Neutrophiles (%)**	60.10%
**Lymphocytes (%)**	30.50%
**NLR**	2.27%
**Eosinophiles (%)**	2.8%
**Basophiles (%)**	< 1%
**Monocytes (%)**	4.90%
**Vitamin D (VitD) (ng/mL)**	30.5 ± 12.3
**CRP (mg/L)**	8.6 ± 25.8
**Calcium (mg/dL)**	9.9 ± 0.6
***Samples Distribution (n* = *712)***	
Swab N (%)	12 (1.7%)
Blood Culture N (%)	15 (2.1%)
Catheter N (%)	3 (0.3%)
Urine N (%)	675 (94.9%)
Sputum N (%)	7 (1%)
***Infection*** ***N (%)***	
*No*	633 (88.9%)
*Yes*	79 (11.1%)

*BMI* body mass index, *HBA1C* hemoglobin A1C, *HDL-C* high-density lipoprotein cholesterol, *LDL-C* low-density lipoprotein cholesterol, *NLR* neutrophils-to-lymphocytes ration, *VitD* vitamin D, *CRP* C-reactive protein

We divided our participants according to HbA1c levels into three groups; non-diabetic, pre-diabetic, and diabetic patients (Table 2). This stratification showed that 39.2% were non-diabetic, 43.8% were pre-diabetic, and 17% were diabetic (Table 2).

**Table 2:** Demographic and biological characteristics of the study participants according to diabetes status

Characteristics	Non-diabetic (*n* = 1,608 – 39.2%)	Pre-diabetic (*n* = 1,795- 43.8%)	Diabetic (*n* = 690 – 17%)	P
**Age (years)**	49.5 ± 16.5	63.0 ± 13.3	65.8 ± 13.2	< 0.001
**Gender N(%)**				
*Female (%)*	957 (59.5%)	783 (43.6%)	375 (54.3%)	< 0.001
*Male (%)*	651 (40.5%)	1,012 (56.4%)	315 (45.7%)	< 0.001
**Infection N(%)**				
*Yes*	28 (9.5%)	34(11.4%)	17 (14.5%)	0.329
*No*	268 (90.5%)	265(88.6%)	100 (85.5%)	
**BMI (kg/m2)**	25.6 ± 3.3	29.5 ± 4.6	31.0 ± 4.9	0.261
**Total Cholesterol (mg/dL)**	187.5 ± 39.2	185.3 ± 44.0	172.4 ± 47.2	< 0.001
**HDL-C (mg/dL)**	55.0 ± 15.2	51.2 ± 15.4	45.0 ± 13.0	< 0.001
**LDL-C (mg/dL)**	109.3 ± 33.2	105.6 ± 36.9	92.1 ± 37.9	< 0.001
**Triglycerides (mg/dL)**	121.4 ± 72.6	150.3 ± 83.3	195.6 ± 175.8	< 0.001
**Neutrophiles (%)**	59.60%	59.90%	62.30%	< 0.001
**Lymphocytes (%)**	31.40%	30.80%	27.50%	< 0.001
**Eosinophiles (%)**	2.80%	2.90%	2.70%	0.135
**Basophiles (%)**	< 1%	< 1%	< 1%	0.002
**Monocytes (%)**	5.60%	5.60%	4.20%	0.008
**NLR N(%)**				
*Normal*	1283 (86%)	1324 (84%)	409 (71%)	< 0.001
*High*	215 (14%)	254 (16%)	164 (29%)	
**Vitamin D (VitD) (ng/mL)**	29.9 ± 12.2	31.1 ± 12.0	30.4 ± 14.1	0.018
**CRP (mg/L)**	4.1 ± 9.3	7.9 ± 22.1	22.5 ± 49.8	< 0.001
**Calcium (mg/dL)**	9.8 ± 0.5	9.9 ± 0.6	9.8 ± 0.7	0.094

*BMI* body mass index, *HB A1C* hemoglobin A1C, *HDL-C* high-density lipoprotein cholesterol, *LDL-C* low-density lipoprotein cholesterol, *VitD* vitamin D, *CRP* C-reactive protein

*P* < 0.05 statistically significant

As expected, diabetic individuals (65.8 ± 13.2 years old) were older than the remaining groups (63 ± 13.3 years old mean age of the pre-diabetic group and 49.5 ± 16.5 years old mean age of the non-diabetic). Gender repartition showed a different pattern in pre-diabetic than diabetic individuals; whereas males were predominantly prediabetic (56.4%, Table 2), and females were diabetic (54.3%, Table 2).

The cholesterol levels were the lowest in the diabetic group (Table 2). In contrast, triglycerides levels were the highest in diabetic patients (Table 2).

The most apparent significant difference between the three groups was the differential CBC, specifically the neutrophils and the CRP levels (Table 2). Diabetic individuals had a significantly higher percentage of neutrophils, NLR ratio, and CRP levels when compared with non and pre-diabetic individuals (*P* < 0.001, Table 2). Interestingly, the individuals with high NLR were mostly diabetic (*P* < 0.001, Table 2).

At the infectious levels, the highest prevalence of infections was observed in the pre-diabetic group (43.8%), and *E. coli* was the leading cause of urinary tract infections (44.2%, *P* > 0.05) (Table 3). Similarly, the distribution of bacterial species did not differ significantly across the study groups (Table 3).

**Table 3:** Distribution of Samples and Infectious agents according to HBA1C levels

	**Non-diabetic (*N* = 296)**	**Pre-diabetic (*N* = 299)**	**Diabetic (*N* = 117)**	P*
**Samples (*n* = 712)**				
Body Fuid	1 (0.1%)	0 (0)	1 (0.1%)	N.S
Swab	4 (0.2%)	4 (0.2%)	4 (0.6%)	
Blood Culture	1 (0.1%)	7 (0.4%)	7 (1%)	
Catheter	0 (0)	0 (0)	1 (0.1%)	
Urine	289 (18%)	287 (16%)	99 (14.3%)	
Sputum	1 (0.1%)	1 (0.1%)	5 (0.7%)	
**Bacteria species (*n* = 79)**				
*E. coli*	18 (34.6%)	23 (44.2%)	11 (21.2%)	N.S
*Klebsiella Pneumoniae*	3 (42.9%)	4 (57.1%)	0 (0)	
*Staphylococcus aureus*	2 (66.7%)	0 (0)	1 (33.3%)	
*Citrobacter koseri*	0 (0)	1 (100%)	0 (0)	
*Enterobacter cloacae*	0 (0)	1 (50%)	1 (50%)	
*Enterococcus spp*	1 (16.7%)	2 (33.3%)	3 (50%)	
*Staphylococcus aureus*	0 (0)	1 (100%)	0 (0)	
*Pseudomonas Aeruginosa*	1 (50%)	0 (0)	1 (50%)	
*Proteus mirabilis*	3 (60%)	2 (40%)	0 (0)	

^*^P for Chi-square test. *N.S* Not significant

Multiple logistic regression was used to determine the risk factors for diabetes in the study participants while adjusting for age, gender, BMI, and triglycerides levels. This analysis showed that age, gender, and triglycerides levels were significant predictors (*P* < 0.001, Table 4). Interestingly, NLR and calcemia status increased the risk of diabetes by 30% (OR = 1.3, *p* < 0.05, Table 4) and 100%, respectively (OR = 2.0, *p* < 0.001, Table 4). On the other hand, female gender was associated with decreased risk of diabetes (OR = 0.7, *p* < 0.035) (Table 4).

**Table 4:** Multiple logistic regression analysis with diabetes

**Variables**	**Diabetes**	
	**OR (95% CI)**	P
Age	1.1 (1.03–1.05)	< 0.001
Gender	0.7 (0.5–0.9)	0.010
BMI	1.7 (0.55–1;5)	0.356
Triglycerides	2.5 (1.9–3.3)	< 0.001
NLR	1.3 (1.0–1.7)	0.05
Calcemia	2.0 (1.3–3.1)	0.001

*BMI* Body mass index, *NLR* neutrophils-to-lymphocytes ratio

An interaction analysis between calcemia and NLR in diabetes was performed. This analysis is used to determine if these two factors might interact and influence the risk of T2D. The analysis has shown that both variables interact significantly to alter the diabetes status (O.R = 1.44, *P* = 0.002, Table 5).

**Table 5:** Interaction analysis between calcemia status and neutrophils to lymphocytes ratio on Diabetes status

**Variables**	**Diabetes**	
	**OR (95% CI)**	P
Age	1.1 (1.03–1.05)	< 0.001
Gender	0.7 (0.5–0.9)	0.010
BMI	1.7 (0.55–1;5)	0.356
Triglycerides	2.5 (1.9–3.3)	< 0.001
NLR *Calcemia	1.44 (1.14–1.89)	0.002

*BMI* Body mass index, *NLR* neutrophils-to-lymphocytes ratio

## Discussion

Compelling evidence confirms that the risk of complications in diabetic patients surges as the level of HbA1c increases [25]. Consequently, this study aimed to examine the effect of HbA1c on various inflammatory and infectious clinical markers. As a summary of our results, the mean HbA1c was 5.9%, and 16.8% of the patients had T2D. Significant correlations between age, gender, triglycerides, NLR, and calcemia were found with T2D. Prevalence of infections was 11.1%, and UTIs caused by *E. coli* were the most common cause of infection. Comparing the diabetic group to the pre-diabetic and control groups, the serum CRP level was significantly higher in the diabetic group. Additionally, compared to the pre-diabetic and control groups, diabetic patients displayed a significantly higher percentage of NLR > 3.

Our data showed that out of 4,093 individuals tested, the mean age of participants was 58.2 years old, and the female and male participants accounted for 55.8% and 44.2%, respectively. Participants mostly came from Mount Lebanon and Beirut regions (45.1% and 41.2%, respectively), and the lipid profile, differentiated complete blood count, VitD, and CRP levels were all in the reference range. Among patients tested for infections from various body sites, 11.1% tested positive.

Apart from age, gender, and triglycerides levels, which are well-known risk factors, the calcemic status significantly increased the risk of diabetes by 30% in our Lebanese population. Interestingly, we did not observe VitD deficiency in opposition to previous studies showing a high prevalence of VitD deficiency in patients with chronic diseases such as insulin resistance, T2D, hypertension, and bacterial infections [26–29]. It is important to note that variable levels of vitD deficiency are found across the MENA region [30]. Among adults aged 18 to 65, the prevalence of individuals with vitD level below 20–25 ng/ml varied across different age groups and countries, including Bahrain, Egypt, Iran, Jordan, KSA, Kuwait, Lebanon, and Syria, between 37 and 96%. Notably, the highest incidence of 96% was documented in a population-based study of 2039 young adults from Jordan [30]. VitD levels vary widely depending on factors such as sun exposure and nutritional habits. It is estimated that sun exposure, latitude, pollution, gender, dietary habits, and lack of government policies account for up to 50% of variations in vitD levels, whereas genetic polymorphisms in the vitD pathway account for less than 5% [31]. Differences among ethnic groups have been noted when studying the association between VitD and insulin resistance [18]. Explaining the lack of VitD deficiency in our population requires further study. VitD levels in Lebanese adults are higher when compared to people in Bahrain and KSA [30]. Between 2011 and 2017, several VitD guidelines were published for the MENA region [32–35], raising awareness and prompting physicians to monitor high-risk populations VitD deficiency and recommend Vit D dietary supplementation.

When adjusting for the age, gender, BMI, and triglycerides level, we found that both calcemia and NLR interacted to increase the risk of T2D significantly. Interaction analysis aims to determine whether the effect of one variable on an outcome is influenced by the presence or absence of another variable. This result implies that the combination of abnormal calcemia and an elevated NLR is associated with a higher risk of T2D. Chronic inflammation, as indicated by a high NLR, could potentially exacerbate insulin resistance and contribute to the development of diabetes, especially when combined with disturbances in calcium levels. In fact, the results of a randomized controlled trial published in 2023 have shown in preoperative diabetic foot patients that NLR and serum calcium could be used as reliable indicators to predict the prognosis of diabetic foot [36]. The mechanism behind hypercalcemia association with T2D is complex and not fully understood. Although hypercalcemia is relatively rare, several cohort studies showed that elevated serum calcium correlated with an increased risk of T2D [37–39]. High levels of calcium in the blood may interfere with b-cell secretory function as well as insulin signaling and glucose uptake in skeletal muscle and adipose tissue [40], ultimately leading to insulin resistance. Various other factors, such as the underlying cause of hypercalcemia, the duration and severity of the condition, and the individual's overall health status, should be considered when determining the mechanism involved. A canadian study on 1 182 healthy subjects investigated the relationship between total serum calcium and fasting serum glucose, insulin level, insulin resistance, and beta-cell function [41]. Female subjects with high calcium levels had the highest concentration of glucose and insulin resistance. In contrast, individuals with low calcium levels had the lowest concentration of glucose and the least insulin resistance [41]. It was hypothesized a long time ago that diabetes and cardiovascular disease are linked by a common defect of divalent cation metabolism, including calcium and magnesium [42]. In vitro studies have shown that increased calcium can affect the affinity of insulin receptor and sensitivity to insulin [43] and the release of insulin from pancreatic islets [44].

A low-grade chronic inflammation is observed in diabetic patients, mostly as the result of high visceral adiposity [45]. In obese individuals, various immune cells infiltrate the adipose tissue and release inflammatory mediators leading to systemic inflammation [46]. The therapeutic targeting of inflammation shows promising results in managing diabetic complications [47]. Growing evidence shows that the chronic low-grade inflammation of diabetic patients is associated with an increased risk of cardiovascular events [48]. In our study, CRP level and percentage of high NLR were respectively 5.5 times and two times higher in the diabetic group compared to the non-diabetic group. CRP and NLR have been used as markers of inflammation and immune system activity to predict disease activity, response to treatment or outcome in diseases such as cancer [49, 50], sepsis [51, 52], and cardiovascular diseases [53, 54].

Together with other clinical factors, these inflammatory markers might help improve the management and prognosis of T2D complications.

Diabetes has been shown to present a risk factor for all kinds of infections [55]. Our data showed that the highest prevalence of infections was noticed in the pre-diabetic group (43%), and *E. coli* caused 44.2% of urinary tract infections. These findings are comparable to a study by Kamelija et al., where *E. coli* was one of the most common pathogens in diabetic patients [56]. In addition, Sonkoue Lambou et al. showed that the prevalence of UTI caused by *E. coli* was significantly higher in people with diabetes compared to nondiabetics [57]. These results are mainly due to the lower levels of psoriasin in people with diabetes [58]. Consequently, this low level will increase the risk of bladder infection due to weakening in the cells’ protective barrier function [58]. In addition, the majority of researchers concluded that there is clinical data suggesting that people with diabetes have a higher prevalence of infectious diseases. For example, people with diabetes consult the physicians significantly more often for respiratory tract infections where *Streptococcus pneumoniae* and influenza virus are the most common respiratory infections associated with this disease. This study also mentioned that diabetic patients are more likely to develop UTIs and tuberculosis than non-diabetic people [59].

There are a few limitations to our cross-sectional study. First, more than 80% of the participants came from Beirut and Mount Lebanon regions, limiting the representations of Beqaa, North and South Lebanon regions and limiting the generalizability of the findings. Another limitation was the limited number of participants with a positive infectious status (*n* = 79), which reduced the statistical power of our analysis and our ability to detect for example, significant association in the distribution of bacterial species across the study groups. Because of the method of participant recruitment, another limitation was the mean age of our sample population (58.2 years old) which is not representative of the mean age of the general Lebanese population, that is much younger. Although this represents a selection bias, studying an older age group allows us to shed light on the dynamic progression of diabetes and the implications of our study for early prevention and intervention.

The prevalence of T2D in Lebanon, as reported by the International Diabetes Federation (IDF) in 2022, is around 8.9% among adults aged 20–79. This estimation has increased in the past two years, as our results indicate. If the reason for this rapid increase lies in part in the sedentary lifestyle and poor diet habits of the population that has been associated with a high level of obesity in Lebanon [60], the culprit is also the unpreceded economic crisis that hit the country in 2019 weakening the healthcare system [61] and thus decreasing the accessibility of the population to T2D screening exams and glycemic-control medications. Early diagnosis of T2D and better health education in the Lebanese people will be essential to limit the heavy burden of its complications. Several studies in Lebanon and the MENA region found that diabetic patients had poor adherence to management and self-care measures [62, 63]. These self-care measures include adherence to medications, monitoring of blood glucose levels, and regular screening for microvascular and macrovascular complications [64]. New objectives for governmental institutions and health providers should be set. Preventive measures should be implemented urgently to reverse this trend. Other than screening and early detection programs, reducing the prevalence of T2D requires a comprehensive approach that addresses multiple factors contributing to the disease. Some efficient interventions and policies include training healthcare providers to address lifestyle factors, providing counseling on healthy behaviors especially for patients with prediabetes; implementing public health campaigns to raise awareness about the risk factors and lifestyle changes associated with the disease; creating environments that encourage physical activity, such as parks and pedestrian-friendly neighborhoods; improving access to fresh, nutritious foods, especially in underserved communities; implementing taxes on sugary beverages, processed foods, and other high-calorie, low-nutrient, products; improving food labeling to provide more precise information about the nutritional content of products; and collaborating with food manufacturers to reformulate products, reduce portion sizes, and offer healthier options. Finally, continuous research and data collection on the causes and epidemiology of T2D will be necessary to inform future evidence-based policies and interventions in Lebanon.

Identifying reliable prognostic markers such as NLR and CRP and understanding the dysregulation of calcium homeostasis will help improve the diagnosis and management of T2D and confirm the importance of new therapeutic targets to treat diabetes. Future research is necessary to understand better the potential role of additional clinical markers to help improve the diagnosis and treatment of T2D. Finally, the associations observed may not necessarily indicate causation. Therefore, extensive experimental studies are needed to determine the cause-consequence relationship between factors such as hypercalcemia and the onset of type 2 diabetes.

## Data Availability

The authors confirms that the data supporting the findings of this study are available within the article.

## Abbreviations

BMI: Body mass index
CRP: C-reactive protein
E. coli: Escherichia coli
HbA1c: Glycated hemoglobin
HPLC: High-performance liquid chromatographic
IDF: International Diabetes Federation
MENA: Middle East and North Africa region
NLR: Neutrophils-to-lymphocytes ratio
T2D: Type 2 diabetes
UTIs: Urinary tract infections
VitD: Vitamin D
